# Brazilian Breast Sculpting: A Standardized Approach to Mastopexy Without Implants

**DOI:** 10.1093/asjof/ojag040

**Published:** 2026-03-02

**Authors:** Jeimeson Costa, Jediael Magalhães Paiva, Guilherme Bastida, Ana Luiza Chagas Maneira, Ana Elisa Kadri, Caroline Wippich

## Abstract

The authors evaluated the feasibility, safety, and patient-reported outcomes of a standardized autologous protocol (“Brazilian Breast Sculpting”) that integrates liposuction, Liacyr flap creation, intramuscular and subcutaneous fat grafting, a muscular sling, and an internal bra. In this prospective case series, 38 patients (mean age 44 years; BMI ≤28) underwent the 6-step protocol between January 2023 and November 2024 and completed at least 12 months of follow-up. Patient-reported outcomes were assessed at 12 months using the Brazilian Portuguese version of the BREAST-Q reduction/mastopexy module. All patients completed the standardized protocol. Postoperative morbidity was low; wound dehiscence occurred in 5 patients (13.2%) and suture extrusion in 7 (18.4%), with all cases managed conservatively. At 12 months, mean BREAST-Q scores (0-100) suggested high satisfaction, particularly for satisfaction with breasts and physical well-being (chest). These preliminary findings suggest that Brazilian Breast Sculpting is a feasible autologous approach to mastopexy without implants with favorable patient-reported outcomes and an acceptable safety profile.

**Level of Evidence:** 4 (Therapeutic) 
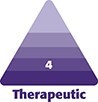

Mastopexy without breast implants remains a significant challenge in aesthetic breast surgery, particularly when addressing upper pole volume deficiency and ensuring sustained shape stability.^1,2^ Although traditional mastopexy techniques effectively correct ptosis, they often fail to provide adequate projection and fullness, especially in the superomedial pole.^3^ The growing demand for natural breast rejuvenation without foreign materials has driven the development of autologous augmentation techniques.^4^

The “Brazilian Breast Sculpting” approach represents a standardized protocol that integrates multiple established autologous techniques—lower dermoglandular flap (Liacyr flap), intramuscular and subcutaneous fat grafting, muscular support sling, and internal bra formation—to address the multifaceted challenges of mastopexy without implants.^5-7^ This preliminary report describes the technique in detail and presents early patient-reported outcomes using the validated BREAST-Q instrument in a prospective case series. The term “Brazilian Breast Sculpting” is used herein as a descriptive designation for this standardized mastopexy protocol, acknowledging the significant contributions of Brazilian plastic surgeons to innovations in aesthetic breast surgery.

## METHODS

### Study Design and Patient Selection

This prospective case series included 38 consecutive patients who underwent mastopexy using the Brazilian Breast Sculpting technique between January 2023 and November 2024 at a private practice in Ribeirão Pires, metropolitan region of São Paulo, Brazil. All patients provided informed consent, and the study was conducted in accordance with the Declaration of Helsinki. Written consent was provided, by which the patients agreed to the use and analysis of their data and clinical images.

Inclusion criteria were the following: (1) age ≥18 years; (2) body mass index (BMI) ≤ 28 kg/m²; (3) postlactational or age-related breast ptosis (Regnault grades I, II, or III); (4) desire for breast rejuvenation without implants; and (5) realistic expectations regarding surgical outcomes.

Exclusion criteria were the following: (1) active smoking; (2) significant or decompensated comorbidities (eg, uncontrolled diabetes, hypertension, autoimmune diseases); (3) history of breast cancer or radiation therapy to the chest; and (4) unrealistic patient expectations.

### Preoperative Assessment

In addition to standard laboratory evaluation, all patients underwent comprehensive preoperative assessment including cardiac evaluation (stress test, Doppler echocardiogram), breast imaging (ultrasound and/or mammography), and vascular Doppler of lower limbs. This comprehensive assessment allowed for identification of potential contraindications and personalization of surgical planning according to anatomical characteristics and specific needs of each patient.

### Surgical Technique: The Brazilian Breast Sculpting Approach

The Brazilian Breast Sculpting technique represents a standardized approach developed to perform mastopexy without the use of breast implants, combining elements of established techniques with specific modifications to optimize shape, projection, and naturalness of results. This approach integrates 6 main technical elements:

1. Axillary and lateral breast liposuction
2. Creation of a Liacyr dermoglandular flap
3. Muscular support sling
4. Internal bra formation
5. Intramuscular fat grafting to the pectoralis major
6. Subcutaneous fat grafting for contour refinement

#### Planning and Markings

Meticulous planning was fundamental to the success of the technique. Markings were made with the patient in an orthostatic position, following established principles:

1. The new point A, which defines the apex of the repositioned nipple-areola complex (NAC), was determined through a bidigital maneuver, similar to the technique described by Pitanguy,^8^ but adapted to the specific needs of the Brazilian Breast Sculpting approach.
2. The preferred incision pattern was Wise (inverted T), which allowed adequate access for all steps of the technique, particularly the creation and positioning of the Liacyr flap.
3. The de-epithelialization area for the creation of the Liacyr dermoglandular flap in the lower pole was carefully marked, maintaining a distance of approximately 2 cm from the areolar margin to preserve vascularization.

This marking approach incorporated elements of the original Liacyr Ribeiro technique,^5^ adapted to the specific context of mastopexy without breast implants, as validated by Souza et al^6^ and Gasperin et al.^7^

#### Infiltration and Liposuction

The procedure began with the infiltration of tumescent solution containing epinephrine (1:200,000) in the lateral regions of the breasts and axillary areas. This step facilitated subsequent liposuction and reduced intraoperative bleeding.

**Figure 1.**
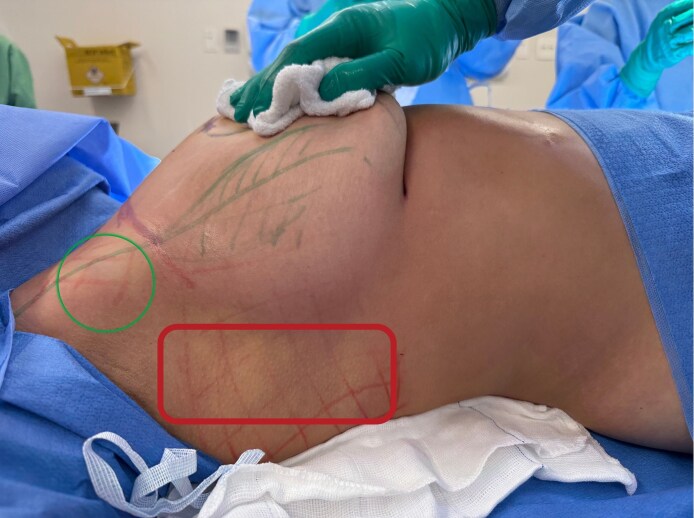
Preoperative markings demonstrating the liposuction zones in the Brazilian Breast Sculpting technique for a 36-year-old female. The circled area indicates the axillary liposuction area, which is harvested to improve lateral contour and reduce axillary fullness. The outlined rectanglular area delineates the lateral breast liposuction area, which provides additional fat for grafting while refining the lateral thoracic contour. Note the surgical markings indicating the planned mastopexy incisions and breast meridian.

After de-epithelialization of the marked area according to the Wise pattern and the design of the lower flap, liposuction of the lateral regions of the breasts and axillary areas was performed (Figure 1). This step served 2 fundamental purposes: (1) Obtaining autologous fat for subsequent fat grafting and (2) defining the lateral contour of the breast, reducing trunk circumference.

Liposuction was performed using 3.5 and 4.0 mm cannulas connected to a standard aspirator. No energy-based devices were used. The aspirated fat was processed by simple decantation, a method that preserves the viability of adipocytes for subsequent grafting, as recommended by recent studies on fat processing for grafting.^9,10^

#### Nipple Areola Complex (NAC) Positioning and Liacyr Flap Creation

The NAC was positioned at the new point A, using a superior-based pedicle. This pedicle choice ensured a reliable blood supply to the NAC, minimizing the risk of ischemic complications.

The creation of the lower dermoglandular flap (Liacyr flap) followed the principles described by Liacyr Ribeiro,^5^ with specific adaptations for the Brazilian Breast Sculpting technique. The typical dimensions of the flap were approximately:

- Width: 5 cm
- Length: 15 cm
- Thickness: 3 cm

**Figure 2.**
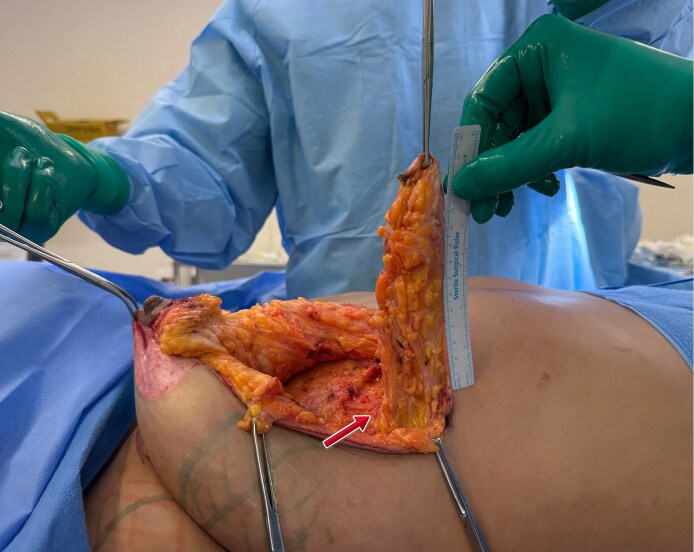
Intraoperative view for a 36-year-old female demonstrating the creation of the Liacyr flap, a deepithelialized inferior pedicle flap with standardized dimensions: 5 cm width, 15 cm length, and 3 cm thickness. The arrow indicates the preserved vascular pedicle, which ensures flap viability. This vascularized flap will be positioned inferior to the muscular sling to provide autologous volume augmentation of the superior and medial breast poles, contributing to natural projection and upper pole fullness without implants.

Preservation of vascularization through the mammary septum was crucial for flap viability, as demonstrated by Gasperin et al.^7^ This flap would serve as an “autologous implant,” providing volume and projection to the upper pole of the breast (Figure 2).

#### Intramuscular Fat Grafting

Before flap fixation, fat grafting was performed in the pectoralis major muscle, a distinctive component of this protocol that differentiates it from conventional mastopexy approaches. The following were used:

- Fine (2.0 mm) and curved cannula
- 3 mL syringes for precise injection control
- Volume of 80-100 mL of decanted fat per side

**Figure 3.**
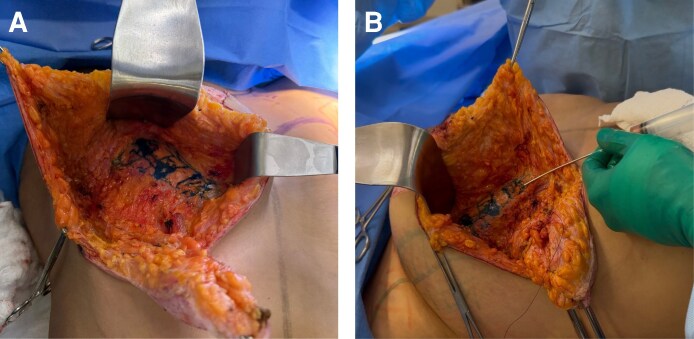
Intramuscular fat grafting technique in the pectoralis major muscle for a 36-year-old female. (A) The target area for intramuscular fat injection is marked in blue, corresponding to the superior and medial regions of the pectoralis major muscle. (B) Intraoperative demonstration of the fan-shaped injection pattern, with autologous fat being deposited in multiple planes within the muscle using a blunt cannula. This technique is designed to enhance volume distribution and improve upper pole contour.

The fat was distributed in a fan-like pattern in multiple layers throughout the muscle, with an emphasis on the superior and medial poles to enhance projection. This intramuscular autologous fat grafting technique followed the principles described by Delay et al^10^ aiming to improve projection and filling of the upper pole of the breast, an area frequently deficient after mastopexy (Figure 3).

#### Muscular Sling Creation

A support sling was created using a portion of the pectoralis major muscle, positioned approximately 6 cm above the inframammary fold (IMF) and about 2-3 cm thick. This step incorporated elements of the technique described by Graf et al,^11^ adapted to the specific context of mastopexy without breast implants.

**Figure 4.**
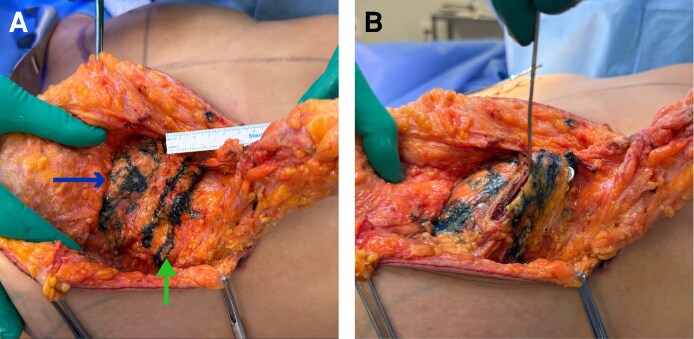
Creation of the muscular support sling, a key structural component of the Brazilian Breast Sculpting technique for a 36-year-old female. (A) Intraoperative planning showing the positioning of the muscular sling approximately 6 cm above the inframammary fold with a width of approximately 3 cm. The horizontal arrow in painel A indicates the visible expansion of the pectoralis major muscle following intramuscular fan-shaped fat grafting. The vertical arrow in panel A shows the intraoperative marking for the muscular support sling placement. (B) Completed muscular support sling after creation, demonstrating the internal structural framework designed to provide breast support and projection without implants.

The tension of the sling was carefully adjusted to allow easy passage of the Liacyr flap underneath it, avoiding vascular constriction or excessive muscular animation, as recommended by Tolazzi et al.^12^ This muscular sling is intended to provide additional structural support and may assist with early maintenance of breast projection and shape; its specific long-term contribution could not be determined within the 12- to 22-month follow-up of this series (Figure 4).

#### Flap Fixation and Internal Bra

The Liacyr flap was transposed superiorly, passing underneath the muscular sling, and fixed to the fascia of the pectoralis major muscle with long-lasting absorbable suture (**Stratafix™ PDS™ 2-0**, Ethicon, Inc., Somerville, NJ, USA). Fixation began laterally and progressed medially, ensuring symmetrical and stable positioning of the flap.

**Figure 5.**
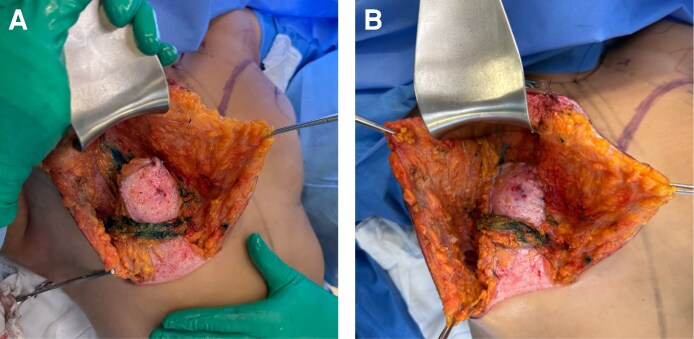
Positioning and fixation of the Liacyr flap for a 36-year-old female. (A) The Liacyr flap is positioned inferior to the muscular support sling. (B) Fixation of the Liacyr flap to the pectoralis major muscle fascia using barbed absorbable suture (Stratafix PDS 2.0; Ethicon Inc, Somerville, NJ). The fixation sequence proceeds from lateral to superior to medial aspects.

Subsequently, the inframammary fold was refixed with 2-0 nylon sutures (Ethicon, Inc., Somerville, NJ), creating an “internal bra” effect as described by Oliveira & Freitas.^13^ This step is conceived as an adjunctive maneuver to provide additional support to the lower pole and potentially delay recurrent ptosis, although its precise long-term impact remains to be established (Figure 5).

#### Assembly and Closure

The patient was positioned sitting at 60 degrees for final breast assembly, resection of excess skin and dermofat tissue, and symmetry verification. This intraoperative evaluation in the sitting position was crucial to ensure optimal aesthetic results, allowing immediate adjustments if necessary.

A 15 Fr Blake drain (Ethicon, Inc., Somerville, NJ) was placed bilaterally to prevent fluid accumulation in the postoperative period. Closure was performed in multiple layers:

- 2-0 nylon for deep planes (Ethicon, Inc., Somerville, NJ)
- 3-0 nylon for middle layer (Ethicon, Inc., Somerville, NJ)
- 3-0 colorless PDS™ for juxtadermal approximation (Ethicon, Inc., Somerville, NJ)
- 4-0 Monocryl™ for intradermal suture (Ethicon, Inc., Somerville, NJ)

**Figure 6.**
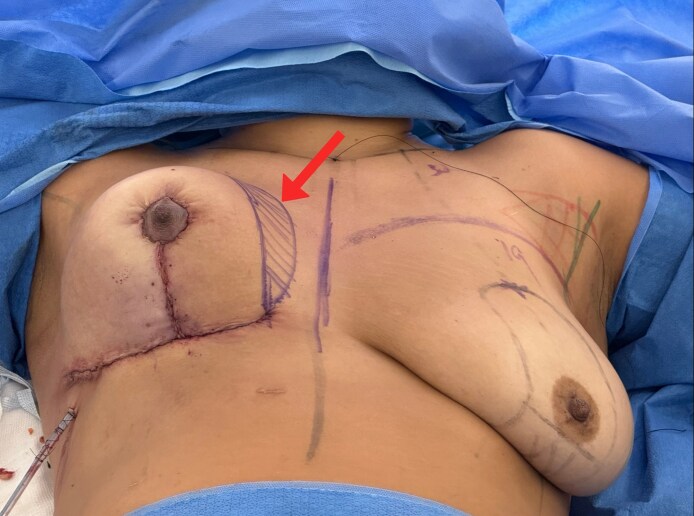
Intraoperative evaluation with the 36-year-old female positioned at 60 degrees following breast assembly and closure. The arrow indicates the subcutaneous fat grafting area, targeting the superior and medial breast contour. This sitting position allows for assessment of breast shape and symmetry after completion of the procedure.

This multilayer closure approach distributed tension and minimized the risk of dehiscence, contributing to aesthetic scars (Figure 6).

#### Subcutaneous Fat Grafting

As a final step, approximately 50-80 mL of fat was injected into the subcutaneous plane using a 2.0 mm cannula to refine the breast contour, with emphasis on filling the superomedial pole. This step followed the principles described by Munhoz et al^14^ for subcutaneous fat grafting, aiming to optimize contour and natural transition between different areas of the breast.

**Figure 7.**
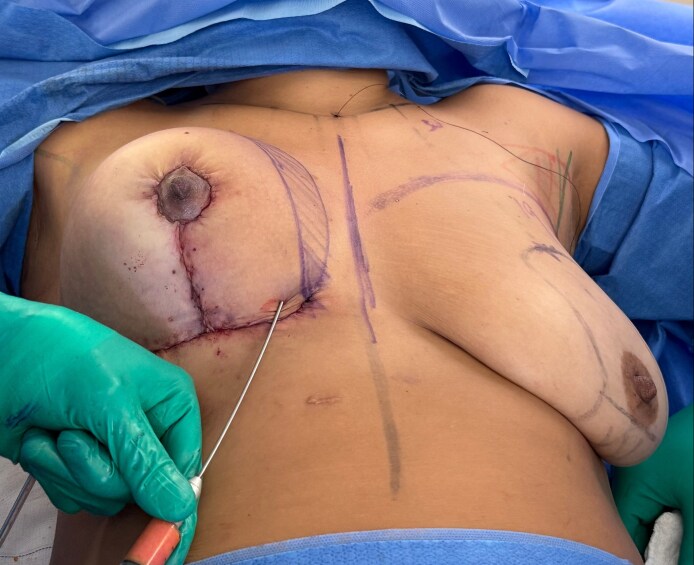
Final subcutaneous fat grafting step with the 36-year-old female positioned at 60 degrees. Approximately 50 to 80 mL of autologous fat is injected into the subcutaneous plane using a 2.0 mm cannula, with emphasis on the superomedial breast contour.

Subcutaneous fat grafting complemented intramuscular autologous fat grafting and the Liacyr flap, providing a harmonious and natural 3-dimensional result, as demonstrated by Papadopoulos et al^3^ in their study on the benefits of fat grafting associated with autoaugmentation (Figure 7).

#### Finalization

The resulting final scar was an inverted T, following the Wise pattern. Surgical glue (Prineo™Skin Closure System, 3 M Health Care, St. Paul, MN) and topical skin adhesive (Dermabond™, Ethicon, Inc., Somerville, NJ) were used to optimize healing and provide additional support to incisions in the immediate postoperative period. A comprehensive video demonstration of the complete surgical technique is provided, available online at www.asjopenforum.com.

### Postoperative Care

Postoperative management was fundamental to optimize results and minimize complications. The standardized protocol included the following:

1. Dressings: The initial dressing was made with Mepilex® Border (Mölnlycke Health Care, Gothenburg, Sweden), which provides an ideal moist environment for healing and minimizes trauma during changes.
2. Support: Continuous use of a compressive surgical bra was indicated for 3 months, providing additional support during the healing and remodeling period.
3. Activity restriction: Patients were advised to avoid physical exertion with the upper limbs for 4 weeks to prevent tension on sutures and optimize healing.
4. Follow-up: Dressings were changed exclusively by the medical team, contributing to the low infection rate. The follow-up protocol included the following:
  - Weekly consultations in the first month
  - Returns at 2, 3, 6 months, and 1 year
5. Scar care: Photoprotection of scars was recommended for 6 months to prevent hyperpigmentation and optimize the final aesthetic result.

### Outcome Assessment

All patients were followed for a minimum of 12 months. Patient-reported outcomes were assessed at 12-month follow-up using the BREAST-Q Reduction/Mastopexy module, administered in Brazilian Portuguese. The BREAST-Q is a validated, breast-specific patient-reported outcome instrument that evaluates satisfaction and quality of life across multiple domains, including satisfaction with breasts, psychosocial well-being, sexual well-being, and physical well-being (Chest).^15^ The questionnaire was administered electronically via Google Forms; electronic administration of the BREAST-Q has been shown to be equivalent to paper-and-pencil formats.^16^ Preoperative BREAST-Q assessment was not performed; only postoperative data at 12-month follow-up were collected. Raw responses were converted to 0-100 scores according to the BREAST-Q Version 2.0 scoring guidelines, with higher scores indicating greater satisfaction.

### Statistical Analysis

Given the preliminary and descriptive nature of this case series, statistical analysis was limited to descriptive statistics. Continuous variables are presented as means with SDs and ranges where appropriate. Categorical variables are presented as frequencies and percentages. No inferential statistical testing or comparative analysis was performed. Data were compiled and analyzed using Microsoft Excel (Microsoft Corporation, Redmond, WA).

## RESULTS

### Patient Demographics

A total of 38 patients underwent the Brazilian Breast Sculpting technique during the study period. Mean age was 44 years (range: 18-67 years). All patients had BMI ≤28 kg/m² (mean: 25.2, range: 23-28) and presented with postlactational or age-related breast ptosis (Regnault grades I, II, or III). Demographic and surgical characteristics are summarized in Table 1.

**Table 1.** Patient Demographics and Surgical Characteristics (*n* = 38)

Characteristic	Mean ± SD	Range
Age (years)	44.0 ± 12.5	18-67
BMI (kg/m^2^)	25.2 ± 1.4	23-28
Ptosis grade, *n* (%)		
Grade I	10 (26.3%)	
Grade II	21 (55.3%)	
Grade III	7 (18.4%)	
Operative time (hours)	4.5 ± 0.4	4.0-5.5
Breast tissue resected (g per breast)	507.5 ± 183.8	140-875
Lipoaspirate volume (mL per side)	320 ± 55	250-450
Intramuscular fat grafting (mL per side)	90 ± 6	80-100
Subcutaneous fat grafting (mL per side)	65 ± 9	50-80
Follow-up (months)	17.0 ± 2.9	12-22

Data are presented as mean ± standard deviation (SD) and range, except for categorical variables presented as *n* (%).

### Surgical Outcomes

All 38 patients underwent the full 6-step protocol of the Brazilian Breast Sculpting technique. Mean operative time was 4.5 hours (range: 4.0-5.5 hours). Mean breast tissue resected was 507.5 ± 183.8 g per breast (range: 140-875 g). Mean volume of lipoaspirate was 320 mL per side (range: 250-450 mL). Mean volume of intramuscular fat grafting was 90 mL per side (range: 80-100 mL), and mean volume of subcutaneous fat grafting was 65 mL per side (range: 50-80 mL). Mean follow-up duration was 17.0 ± 2.9 months (range: 12-22 months), allowing for consistent evaluation of aesthetic outcomes, complications, and satisfaction levels.

### Complications

No major complications were observed. Specifically, there were no cases of seroma, hematoma, clinical fat necrosis, or nipple-areola complex (NAC) necrosis. Minor, clinically manageable complications occurred in a subset of patients. Five patients (13.2%) developed a small wound dehiscence localized at the junction between the vertical and horizontal limbs of the inverted-T scar; all were managed conservatively with topical collagenase ointment and routine dressings, with complete resolution and no need for surgical revision. In 7 patients (18.4%), focal extrusion of nonabsorbable sutures was noted, and these were removed in the clinic by the surgical team, without compromising scar quality or the overall aesthetic outcome. The absence of major complications and the manageable nature of minor wound-healing problems are likely related, at least in part, to strict preoperative patient selection, including the exclusion of active smokers and individuals with major comorbidities such as uncontrolled hypertension, diabetes, obesity, and other significant systemic diseases.

### Patient Satisfaction

**Figure 8.**
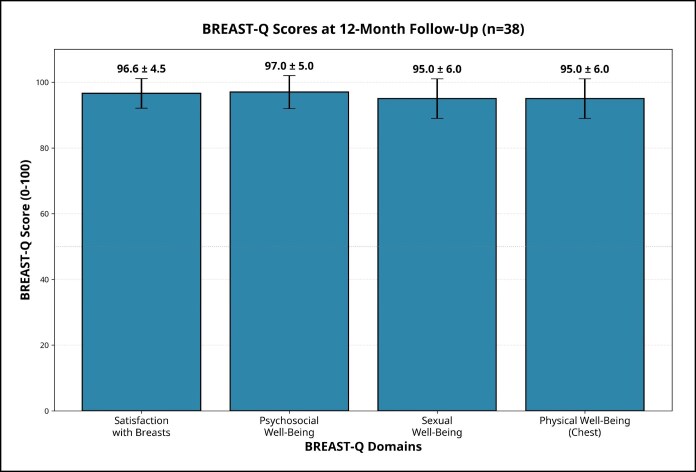
BREAST-Q patient-reported outcome measures at 12-month follow-up (n = 38). Mean scores with standard deviations are shown for 4 domains: satisfaction with breasts (96.6 ± 4.5), psychosocial well-being (97.0 ± 5.0), sexual well-being (95.0 ± 6.0), and physical well-being (chest) (95.0 ± 6.0). Scores range from 0 to 100, with higher scores indicating better outcomes. Error bars represent standard deviation.

At 12 months (*n* = 38), mean BREAST-Q scores (0-100) were satisfaction with breasts, 96.6 ± 4.5; psychosocial well-being, 97.0 ± 5.0; sexual well-being, 95.0 ± 6.0; and physical well-being (chest), 95.0 ± 6.0** (Figure 8). These preliminary results suggest high levels of patient satisfaction across all evaluated domains.

### Representative Clinical Cases

**Figure 9.**
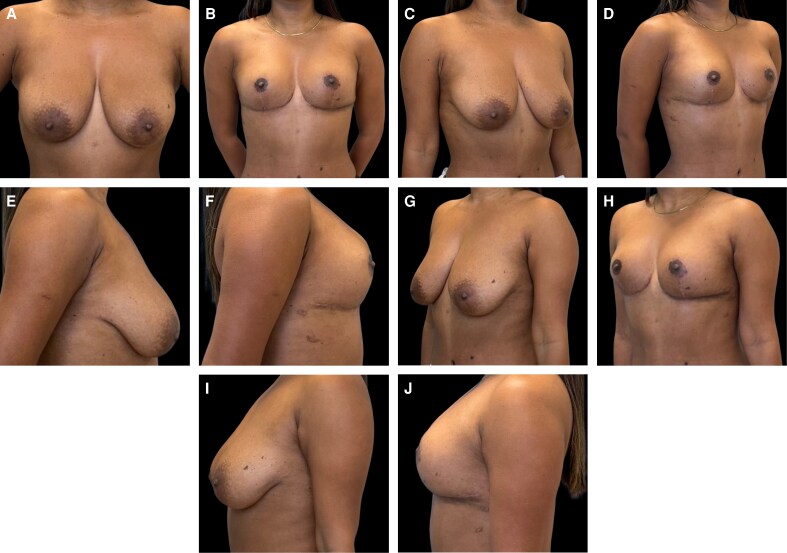
Representative case of a 35-year-old female with one previous pregnancy presenting with breast ptosis and skin laxity. Preoperative views: (A) frontal, (C) right oblique, (E) right lateral, (G) left oblique, and (I) left lateral. Postoperative views at 12 months: (B) frontal, (D) right oblique, (F) right lateral, (H) left oblique, and (J) left lateral.

**Figure 10.**
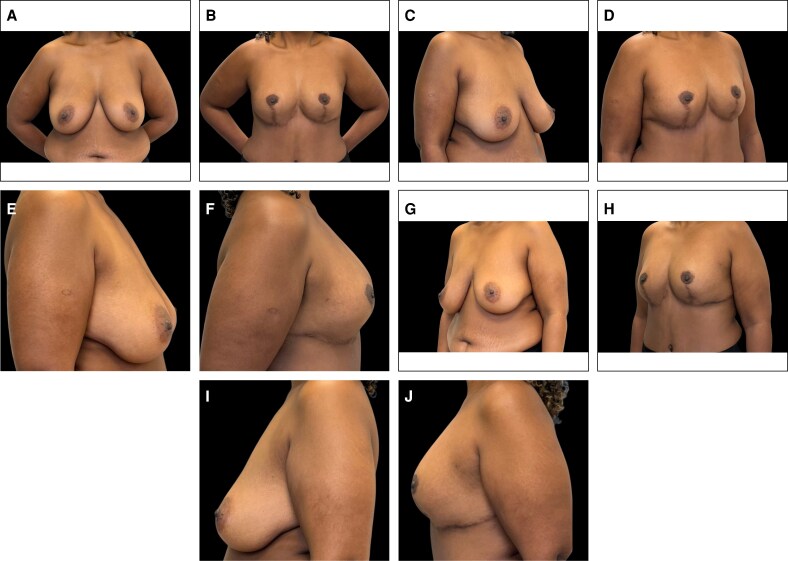
Representative case of a 36-year-old female with one previous pregnancy and 30 kg weight loss following dietary modification, presenting with breast ptosis and skin laxity. Preoperative views: (A) frontal, (C) right oblique, (E) right lateral, (G) left oblique, and (I) left lateral. Postoperative views at 12 months: (B) frontal, (D) right oblique, (F) right lateral, (H) left oblique, and (J) left lateral.

**Figure 11.**
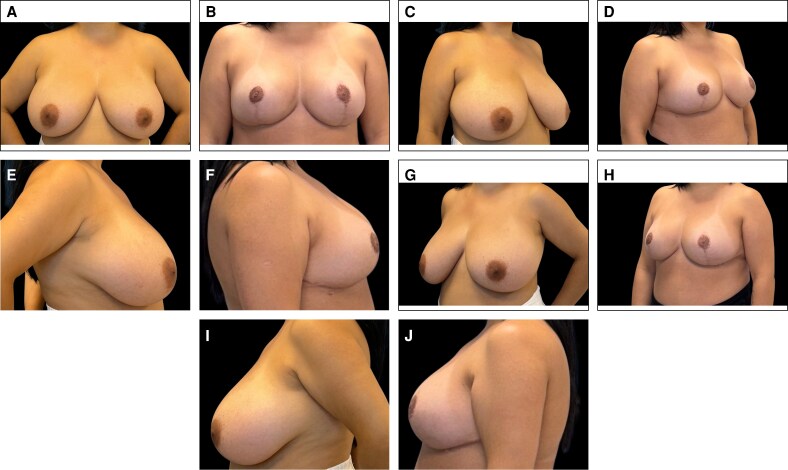
Representative case of a 35-year-old female presenting with breast hypertrophy. Preoperative views: (A) frontal, (C) right oblique, (E) right lateral, (G) left oblique, and (I) left lateral. Postoperative views at 12 months: (B) frontal, (D) right oblique, (F) right lateral, (H) left oblique, and (J) left lateral.

Representative clinical cases demonstrating the aesthetic outcomes achieved with the Brazilian Breast Sculpting technique are illustrated in Figures 9-11. These cases show preoperative and 12-month postoperative views, demonstrating the technique's ability to address breast ptosis, enhance upper pole projection, and achieve natural-appearing breast contour without the use of implants.

## DISCUSSION

The Brazilian Breast Sculpting approach combines various autologous techniques—lower dermoglandular flap, intramuscular and subcutaneous fat grafting, muscular sling, and internal bra—in a standardized protocol for mastopexy without breast implants. The rationale behind this combination is to use the Liacyr flap for central projection and upper pole filling, reinforced by the muscular sling and internal bra for structural support, although fat grafting adds volume and refines contour, particularly in transition areas and the upper pole, frequently deficient after conventional mastopexy. This multifaceted approach aims to simultaneously address breast ptosis, lack of volume (especially in the upper pole), and the need for internal structural support, offering a potential alternative for patients who wish to avoid breast implants.

From a patient-reported outcome perspective, our 12-month BREAST-Q scores were in the mid-90 seconds across all core domains [satisfaction with breasts, psychosocial well-being, sexual well-being, and physical well-being (chest)]. Although direct comparison with other series is limited by differences in patient selection and surgical indications, these values are broadly consistent with prior Brazilian and international studies using the Reduction/Mastopexy module, which have demonstrated substantial improvements in satisfaction and quality of life after breast reduction surgery. In particular, Andrade et al^17^ reported significantly higher BREAST-Q scores in Brazilian women who underwent reduction mammaplasty compared with women with untreated breast hypertrophy, reinforcing the ability of the instrument to detect clinically meaningful gains after breast surgery. Although direct pre–post comparisons were not possible in this cohort, the very high postoperative BREAST-Q scores are numerically comparable to those reported in established breast reduction cohorts, suggesting that this protocol without implants can achieve similarly favorable levels of patient-reported satisfaction. Although the BREAST-Q does not specifically quantify breast shape parameters such as upper pole projection or base width, the very high postoperative scores suggest that patients perceived the overall aesthetic and functional outcomes as favorable.

Our rigorous postoperative protocol, including exclusive dressing changes by the medical team and standardized follow-up, likely contributed to the low complication rates observed. The minor incidence of wound dehiscence (13.2%) and suture extrusion (18.4%) were managed conservatively without compromising final outcomes, consistent with rates reported in the literature for similar techniques.^18-20^

The use of permanent sutures for internal bra formation and inframammary fold fixation warrants discussion. Although permanent sutures carry theoretical risks of palpability and extrusion, we observed focal suture extrusion in 18.4% of patients, which were easily managed with office-based removal without compromising aesthetic outcomes. In our preliminary experience, permanent fixation appears to offer a scaffold that may assist with medium-term shape maintenance; however, the balance between improved support and the risk of suture palpability or extrusion requires confirmation with longer follow-up and comparative studies. Consistent with prior authors, we believe that adequate resection of excess inferior and lateral parenchyma remains central to long-term stability; the muscular sling and inframammary fold fixation are conceived as adjunctive maneuvers rather than the sole determinants of shape maintenance. The current follow-up of 12-22 months should be interpreted as medium-term; we cannot yet draw firm conclusions about true long-term stability.

### Limitations and Future Directions

As a preliminary prospective case series from a single surgeon's practice, this study has inherent limitations that should be acknowledged. The absence of a control group precludes direct comparison with other mastopexy techniques or with mastopexy combined with implants. The single surgeon design, while ensuring technical consistency, limits assessment of technique reproducibility across different practitioners and surgical settings. The combination of multiplane fat grafting, dermoglandular flap transposition, and muscular manipulation likely entails a learning curve that was not formally assessed in this preliminary series, and reproducibility by other surgeons remains to be determined. The follow-up period of 12-22 months, though adequate for preliminary assessment of safety and patient satisfaction, may not fully capture long-term outcomes such as late ptosis recurrence or progressive fat resorption. Additionally, the BREAST-Q was administered only postoperatively; preoperative baseline assessment would have strengthened our ability to quantify the magnitude of improvement attributable to surgery. In addition, no standardized anthropometric or blinded photographic assessment (eg, serial sternal notch-to-nipple measurements) was performed, which limits objective evaluation of long-term shape stability. Finally, the descriptive nature of the statistical analysis, appropriate for this preliminary report, does not permit comparative or inferential conclusions.

Future investigations incorporating longer follow-up, multicenter collaboration, preoperative and postoperative patient-reported outcome assessment, and comparative study designs will be valuable in more definitively establishing the efficacy, durability, and relative merits of the Brazilian Breast Sculpting technique.

## CONCLUSIONS

The Brazilian Breast Sculpting technique represents a standardized and multifaceted approach to mastopexy without breast implants, combining Liacyr dermoglandular flap, muscular sling, internal bra, and fat grafting in multiple planes. Preliminary results at 12 months suggest high patient satisfaction across multiple BREAST-Q domains and an acceptable safety profile. The integration of multiple autologous techniques in a standardized protocol may offer a structured, stepwise approach to simultaneously addressing the main challenges of mastopexy without breast implants: breast ptosis, lack of volume (especially in the upper pole), and the need for internal structural support. Long-term follow-up will be necessary to assess the durability of these results beyond the 12- to 22-month observation period reported here. The technique may provide a promising autologous alternative for patients who wish to avoid breast implants, either in primary surgery or secondary after explantation, meeting the growing demand for natural results without breast implants.

## References

[1] Graf RM, Reis de Araujo LR, Rippel R, Graça Neto L, Pace DT, Biggs T. Reduction mammaplasty and mastopexy using the vertical scar and thoracic wall flap technique. Aesthetic Plast Surg. 2003;27 6–12. doi: 10.1007/s00266-002-0111-5 12687296

[2] Calobrace MB, Gabriel A. Mastopexy with autoaugmentation and fat transfer. Clin Plast Surg. 2021;48:17–32. doi: 10.1016/j.cps.2020.09.008 33220902

[3] Papadopoulos S, Colpaert SDM, Goulis DG, et al Fat grafting and auto-augmentation mastopexy after breast implant removal: technique and evaluation of outcomes using BREAST-Q. Aesthet Surg J. 2021;41:NP388–NP401. doi: 10.1093/asj/sjaa347 33300983

[4] Hönig JF, Frey HP, Hasse FM, Hasselberg J. Autoaugmentation mastopexy with an inferior-based pedicle. Aesthetic Plast Surg. 2010;34:447–454. doi: 10.1007/s00266-010-9471-4 19225831 PMC2910896

[5] Ribeiro L. A new technique for reduction mammaplasty. Plast Reconstr Surg. 1975;55:330–334. doi: 10.1097/00006534-197555030-00010 1118493

[6] Souza AA, Fajwichow L, Ferreira AA, Simão TS, Pitol DN, Máximo FR. Avaliação das técnicas de mamoplastia quanto a sua influência tardia na distância do complexo areolopapilar ao sulco inframamário. Rev Bras Cir Plást. 2011;26:664–669. doi: 10.1590/S1983-51752011000400022

[7] Gasperin BDM, Pavelecini M, Possamai LM, Freitas Neto FM, Bins Ely P. Viabilidade do uso de retalhos de Liacyr dos tipos I e III em mastopexias com implantes em pacientes com incisão prévia no sulco inframamário. Rev Bras Cir Plást. 2018;33:28–30. doi: 10.5935/2177-1235.2018RBCP0035

[8] Pitanguy I. Breast hypertrophy. In: Goldwyn RM, ed. Plastic and Reconstructive Surgery of the Breast. Little, Brown and Company; 1976:223–266.

[9] Coleman SR. Structural fat grafting: more than a permanent filler. Plast Reconstr Surg. 2006;118:108S–120S. doi: 10.1097/01.prs.0000234610.81672.e7 16936550

[10] Delay E, Garson S, Tousson G, Sinna R. Fat injection to the breast: technique, results, and indications based on 880 procedures over 10 years. Aesthet Surg J. 2009;29:360376. doi: 10.1016/j.asj.2009.08.010 19825464

[11] Graf RM, Auersvald A, Bernardes A, Biggs TM. Reduction mammaplasty and mastopexy with shorter scar and better shape. Aesthet Surg J. 2000;20:99–106. doi: 10.1067/maj.2000.106471

[12] Tolazzi ARDO, Graft RM, Freitas Rs, Cruz GAO. Avaliação da influência da cinta muscular peitoral na sustentação do retalho dermolipoglandular mamário após mamoplastia vertical. Rev Bras Cir Plást. 2010;25:141–152.

[13] Oliveira M, Freitas A. Long-term results of internal brain mastopexy without breast implants: a three-year follow-up study. Aesthet Surg J. 2019;39:412–418.

[14] Munhoz AM, Maximiliano J, de Azevedo Marques Neto A, et al Zones for fat grafting in hybrid breast augmentation: standardization for planning of fat grafting based on breast cleavage units. Plast Reconstr Surg. 2022;150:782–795. doi: 10.1097/PRS.0000000000009605 35877935

[15] Pusic AL, Klassen AF, Scott AM, Klok JA, Cordeiro PG, Cano SJ. Development of a new patient-reported outcome measure for breast surgery: the BREAST-Q. Plast Reconstr Surg. 2009;124:345–353. doi: 10.1097/PRS.0b013e3181aee807 19644246

[16] Fuzesi S, Cano SJ, Klassen AF, Atisha D, Pusic AL. Validation of the electronic version of the BREAST-Q in the Army of Women study. Breast. 2017;33:44–49. doi: 10.1016/j.breast.2017.02.015 28279888 PMC5551502

[17] Andrade AC, Veiga DF, Aguiar IC, Juliano Y, Sabino-Neto M, Ferreira LM. Outcomes analysis of breast reduction in Brazilian women using the BREAST-Q® questionnaire: a cross-sectional controlled study. Clinics (Sao Paulo). 2018;73:e313. doi: 10.6061/clinics/2018/e313 29924186 PMC5996439

[18] Graf RM, Biggs TM. In search of better shape in mastopexy and reduction mammaplasty. Plast Reconstr Surg. 2002;110:309–317. doi: 10.1097/00006534-200207000-00053 12087273

[19] Erfon M, Tenna S, Persichetti P. Autoaugmentation mastopexy: a versatile technique for aesthetic improvement of the breast. Aesthet Surg J. 2018;38:487–494.

[20] Colwell AS, Breuing KH. Improving shape and symmetry in mastopexy with autologous or cadaveric dermal slings. Ann Plast Surg. 2008;61:138–142. doi: 10.1097/SAP.0b013e31815bfe7c 18650604

